# A Novel Prognostic Biomarker of Luminal Breast Cancer: High CD39 Expression Is Related to Poor Survival

**DOI:** 10.3389/fgene.2021.682503

**Published:** 2021-06-18

**Authors:** Xiaojian Ni, Wenze Wan, Jingjing Ma, Xinyou Liu, Bohao Zheng, Zhixian He, Weige Yang, Lihong Huang

**Affiliations:** ^1^Department of General Surgery, Zhongshan Hospital, Fudan University, Shanghai, China; ^2^Cancer Center, Zhongshan Hospital, Fudan University, Shanghai, China; ^3^Nanjing Maternity and Child Health Care Hospital, Women’s Hospital of Nanjing Medical University, Nanjing, China; ^4^Department of General Surgery, Xiamen Branch, Zhongshan Hospital, Fudan University, Xiamen, China; ^5^Department of General Surgery, Affiliated Hospital of Nantong University, Nantong, China; ^6^Department of Biostatistics, Zhongshan Hospital, Fudan University, Shanghai, China

**Keywords:** nucleoside triphosphate diphosphohydrolase-1, breast cancer, The Cancer Genome Atlas, prognosis, tumor immunology

## Abstract

**Background:**

CD39 is one of the functional surface markers for T regulatory cells, the prognostic role and immune-related effects of CD39 in luminal breast cancer (BC) patients has not been evaluated yet. The aim of the current study was to explore the association between CD39 expression and clinic pathological characteristics and the prognosis in luminal BC patients.

**Methods:**

Clinical information and RNA-sequencing (RNA-Seq) expression data were extracted from The Cancer Genome Atlas (TCGA). Patients were divided into a high or low CD39 expression group by the optimal cutoff value (4.18) identified from the receiver operating characteristic curve analysis. The relationships between CD39 expression and clinic pathological features were evaluated by the corresponding statistical tests. Survival analyses were applied to evaluate the overall survival between the high and low CD39 expression groups in luminal BC. Furthermore, Gene Expression Omnibus datasets were used for external data validation. Gene set enrichment analysis (GSEA) was also performed, and CIBERSORT was used to analyze the immune cell populations.

**Results:**

Analysis of 439 cases of tumor data showed that CD39 was overexpressed in luminal BC. The multivariable analysis suggested that CD39 expression was an independent prognostic factor for luminal BC patients. GSEA suggested that CD39 might play an important role in luminal BC progression through immune regulation. Analysis of immune cell patterns revealed high CD39 expression correlated to a higher proportion of CD8^+^ T cells and M2 macrophages.

**Conclusion:**

This study demonstrates that CD39 expression correlates with the prognosis of luminal BC through TCGA database mining. Further studies are warranted further to elucidate this potential novel therapeutic strategy for BC.

## Introduction

Breast cancer (BC) is the most familiar malignant neoplasm in women worldwide, with multiple molecular subtypes (Siegel and Miller, 2020). Tumor size, histological grade, lymph node stage, and hormone receptor status were the common risk factors of BC (Chia et al., 2008; Soerjomataram et al., 2008). However, BC’s propensity of giving rise to distant metastases also depends on the molecular subtype. Luminal A-like tumors were defined as estrogen receptor-positive (ER+), human epidermal growth factor receptor 2 negatives (HER2−), progesterone receptor (PR) ≥ 20%, Ki67 < 14% which has “low” recurrence risk assessed by gene assays (Goldhirsch et al., 2013). Luminal B-like tumors were defined as ER+, HER2−, and at least one of the following: PR negative < 20%, Ki67 ≥ 20%, and which has “high” recurrence risk based on gene assays. It was reported that the most common subtype of BC is the ERα+ subtype (luminal A or luminal B), which comprises 80% of all BC (Metzger-Filho et al., 2013). Bone is the most common metastatic site in luminal subtypes (Kennecke et al., 2010). Although BC has a better prognosis and a higher overall survival (OS) rate, especially the luminal subtypes (Kennecke et al., 2010; Wu et al., 2017), it remains a challenge to reduce BC’s bone metastasis and mortality. Hence, identifying novel molecular signature to predict BC’s prognosis, especially the most common luminal ERα+ subtype, is of great importance.

Ectonucleoside triphosphate diphosphohydrolase 1 (ENTPD1/CD39) is not the only ATP-degrading enzyme that is expressed by tumor cells; for example, nucleoside triphosphatase, cancer-related (NTPCR) is also expressed by some tumors which can bind to extracellular ATP and then convert it to adenosine (Moesta et al., 2020). Its identification derives from studies on genetics, biochemistry, pharmacology, and immunology revealing the broad immunosuppressive effects of adenosine (Wolberg et al., 1975; Huang et al., 1997; Ohta and Sitkovsky, 2001; Ohta et al., 2006). CD39 is widely expressed in immune cells and non-immune cells (Lei et al., 2015). CD39 is also detected in some tumor cells, and intratumoral immune cells demonstrate elevated CD39 expression (Moesta et al., 2020). CD39 dysregulation has been proved to be associated with many malignancies, including melanoma (Dzhandzhugazyan et al., 1998), leukemia (Pulte et al., 2011), pancreatic cancer (Künzli et al., 2007), ovarian cancer (Häusler et al., 2011), and colon cancer (Künzli et al., 2011). In BC patients’ specimens, CD39^+^CD8^+^ T cells were expressed in tumors or metastatic lymph nodes rather than non-invaded lymph nodes or peripheral blood (Canale et al., 2018). However, the prognostic role and immune-related effects of CD39 in luminal BC patients has not been evaluated yet.

Here, for the first time, we explored whether CD39 is a factor affecting luminal BC’s prognosis. We explored the association between CD39 and clinic features and the prognosis using The Cancer Genome Atlas (TCGA)-BRCA level 3 data, and Gene Expression Omnibus (GEO) dataset GSE86166 was applied as external test data to validate the prognostic performance. Moreover, GSE 45827 was used to validate CD39 high expression in tumor tissue. Subsequently, we performed gene set enrichment analysis (GSEA) to explore the related regulatory network signaling pathways of CD39 in BC. To investigate the influence of CD39 on the tumor microenvironment, CIBERSORT was employed to analyze tumor-infiltrating immune cells (TIICs) associated with CD39 expression.

## Materials and Methods

### Patient Samples in TCAG and GEO Datasets

The RNA-sequencing (RNA-Seq) expression data and the clinical data of luminal BC patients were acquired from TCGA^1^. The analysis process was conducted using the RNA-Seq by Expectation-Maximization (RSEM) expression values. GEO datasets were acquired from the GEO database^2^.

### Gene Set Enrichment Analysis

To investigate the influence of CD39 on pathway-level changes in BC tissues, we conducted GSEA to explore whether *a priori* defined set of genes demonstrated significant difference in expression between the constructed high and low CD39 expression groups (the grouping method is shown in the section “Statistical Analysis”) in the TCGA cohort. The pathway with a normal *P*-value < 0.01 and a false discovery rate (FDR) < 0.01 was considered to be significantly enriched.

### Analysis of Tumor-Infiltrating Immune Cells

CIBERSORT, a computational method developed by Newman et al. (2015), was employed to analyze the TIIC fractions of BC samples based on TCGA. The standardized processed data set of gene expression was uploaded in the CIBERSORT website^3^. Monte Carlo sampling was conducted for the deconvolution of each sample to improve the algorithm’s accuracy. Only samples with a CIBERSORT *P* < 0.05 were enrolled in analysis. Moreover, the Wilcoxon rank-sum tests were performed for the comparisons of the TIIC fractions between the constructed CD39 expression high and low groups.

### Statistical Analysis

All statistical analyses and plots were performed using R (v.3.5.1). A comparison of targeted gene expression in BC and normal tissues was conducted using the Wilcoxon rank-sum test using GEO datasets (GSE45827), which included 141 BC patients, and visualized in box plots. The Kruskal–Wallis test and Wilcoxon rank-sum tests were used to evaluate the relationships between CD39 and clinicopathological features. Time-dependent receiver operating characteristic (ROC) curve analysis was used to determine the optimal cutoff value of CD39 in prognosis analysis in both training and validation datasets. Patients were then divided into two groups (i.e., high expression and low expression) in terms of cutoff value. We used a Chi-square test or Fisher’s exact test to determine the link between CD39 groups and clinicopathological features. Kaplan–Meier method was used to plot the survival curves and the difference was compared by the Log-rank test. The relevant clinicopathological characteristics were screened by univariate analysis, and multivariable analysis was then performed to investigate association between CD39 and the survival of luminal BC patients. We evaluated the prediction performance of the constructed CD39 groups using an external GEO dataset (accession ID: GSE86166), which included the expression levels of 247 luminal patients. Additionally, the comparisons of three adenosine receptors (ARs) (ADORA2A, ADORA2B, and ADORA3) in BC and normal tissues were analyzed based on GSE45827 dataset using the Wilcoxon rank-sum test and visualized in box plots, Pearson correlation coefficients between three ARs and CD39 were also estimated, separately.

**FIGURE 1:**
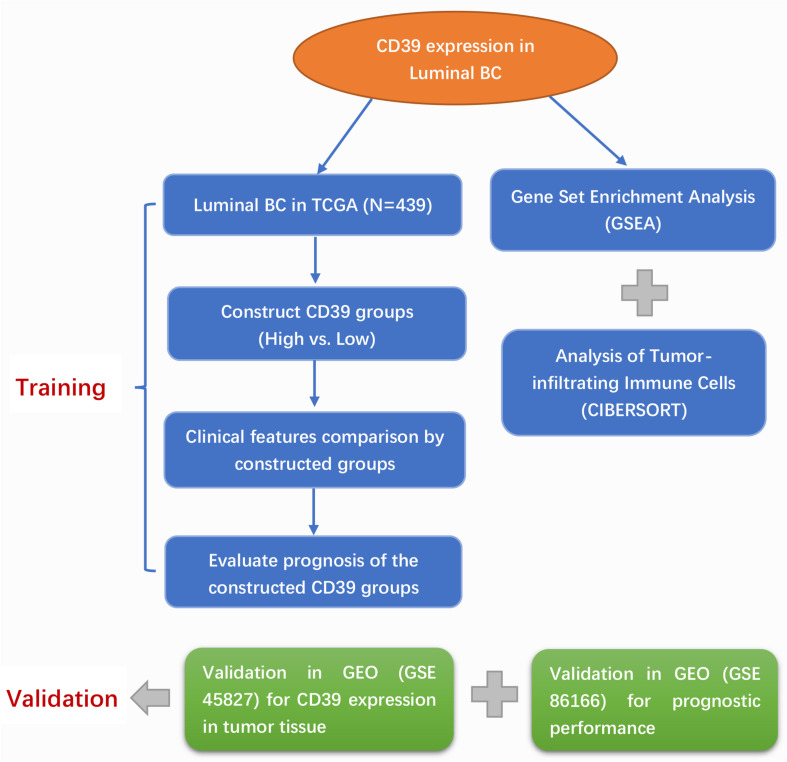
Flow chart indicating the study design of the present work.

The workflow of our analysis is presented in Figure 1.

## Results

### Clinic Pathological Features of Patients

The analysis was performed in the TCGA-BRCA level 3 data. The clinic pathological features, including sample type, histological type, clinical stage, TNM stage, lymph node status, ER, PR, HER-2, molecular subtype, and vital status, are summarized in Table 1. In the investigated population (*N* = 439), most luminal BC patients were not Hispanic or Latino (74.72%), and nearly half of the BC patients were older than 60 years (47.61%). In terms of BC subtypes, 66.51% of the 439 luminal BC patients were classified as infiltrating ductal carcinoma, and 23.01% were infiltrating lobular carcinoma. Furthermore, 97.95% of patients were ER positive. In terms of BC staging, 26.65% were T1, 56.04% were T2, 85.19% were M0, 47.15% were N0, 33.03% were N1, 17.77% were stage I and 56.26% were stage II.

**TABLE 1:** Clinical characteristics of TCGA-BRCA level 3 cohort.

Characteristics	Variable	Numbers of cases (%)
CD39	Low	213 (48.52)
	High	226 (51.48)
Ethnicity	HISPANIC OR LATINO	19 (4.33)
	NOT HISPANIC OR LATINO	328 (74.72)
	NA	89 (20.27)
	Not evaluated	2 (0.46)
	Unknown	1 (0.23)
Age	<60	230 (52.39)
	≥60	209 (47.61)
Sex	Female	434 (98.86)
	Male	5 (1.14)
Histological type	Infiltrating ductal carcinoma	292 (66.51)
	Infiltrating lobular carcinoma	101 (23.01)
	Other	46 (10.48)
ER	Negative	9 (2.05)
	Positive	430 (97.95)
PR	Indeterminate	1 (0.23)
	Negative	64 (14.58)
	Positive	374 (85.19)
HER2	Negative	437 (99.54)
	Positive	2 (0.46)
T classification	T1	117 (26.65)
	T2	246 (56.04)
	T3	63 (14.35)
	T4	12 (2.73)
	TX	1 (0.23)
M classification	M0	374 (85.19)
	M1	4 (0.91)
	MX	59 (13.44)
	NA	2 (0.46)
N classification	N0	207 (47.15)
	N1	145 (33.03)
	N2	53 (12.07)
	N3	29 (6.61)
	NX	5 (1.14)
Stage	I	78 (17.77)
	II	247 (56.26)
	III	103 (23.46)
	IV	4 (0.91)
	NA	4 (0.91)
	X	3 (0.68)
Vital status	Alive	401 (91.34)
	Dead	38 (8.66)

*NA, not available.*

### CD39 Expression in Luminal BC

**FIGURE 2:**
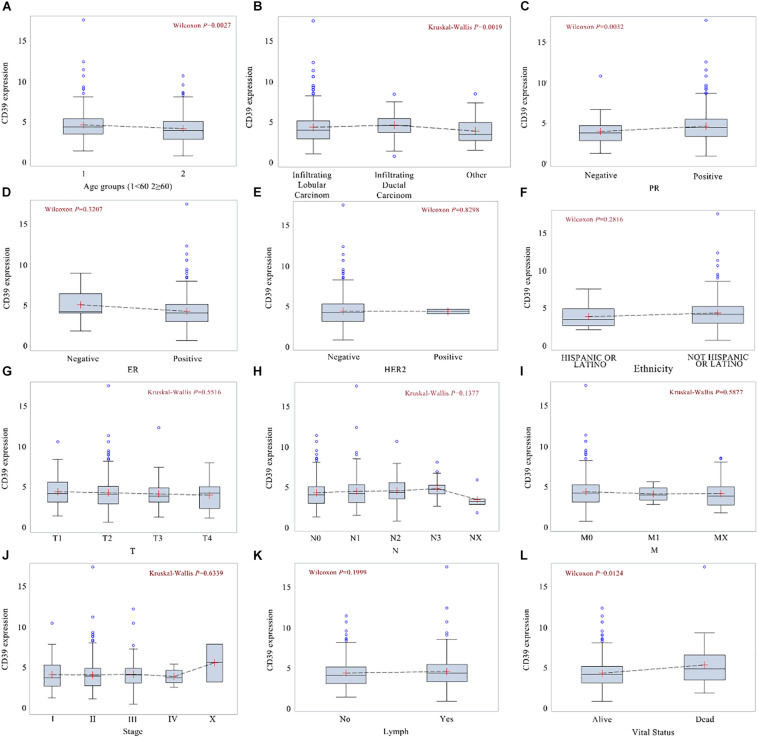
Differences in CD39 expression were shown in boxplots according to patient age **(A)**, histological type **(B)**, PR **(C)**, ER **(D)**, HER-2 **(E)**, molecular subtype **(F)**, T classification **(G)**, N classification **(H)**, M classification **(I)** clinical stage **(J)**, lymph node status**(K)**, and vital status **(L)**.

CD39 expression differences were depicted in boxplots according to patient age (Figure 2A, *P* = 0.0027), histological type (Figure 2B, *P* = 0.0019), PR (Figure 2C, *P* = 0.0032), ER (Figure 2D, *P* = 0.3207), HER2 (Figure 2E, *P* = 0.8298), ethnicity (Figure 2F, *P* = 0.2816), T classification (Figure 2G, *P* = 0.5516), N classification (Figure 2H, *P* = 0.1377), M classification (Figure 2I, *P* = 0.5877), clinical stage (Figure 2J, *P* = 0.6339), lymph node (Figure 2K, *P* = 0.1999), and vital status (Figure 2L, *P* = 0.0124).

**FIGURE 3:**
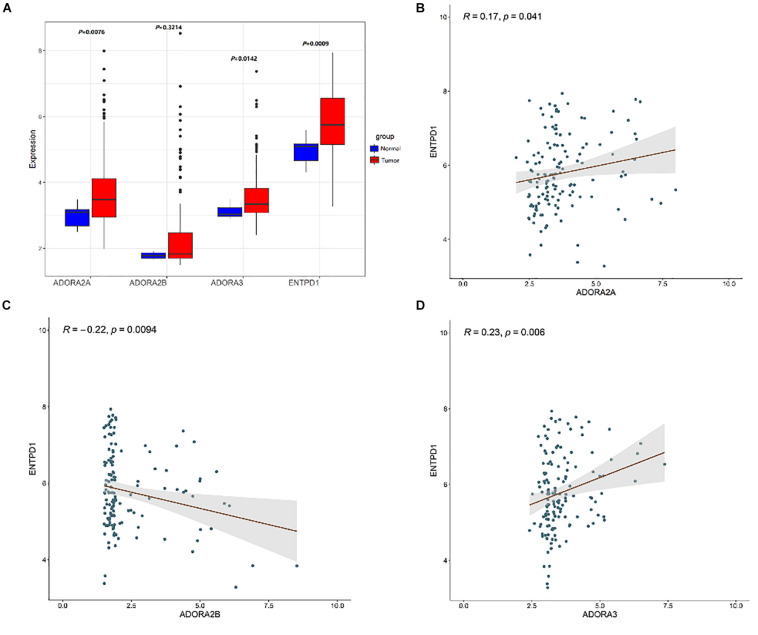
**(A)** Box plots exhibiting the ASORA2A, ADORA2B, ASORA3, and CD39 (ENTPD1) expression in tumor (red) and normal tissues (blue) based on GSE45827. Expression correlation of ENTPD1 with ADORA2A **(B)**, ADORA2B **(C)**, ADORA3 **(D)** in GSE45827.

Compared to normal breast tissues, CD39 was higher in BC in the microarray GSE45827 dataset (*P* = 0.0009, Figure 3).

### The Association Between CD39 Expression and Clinicopathological Features in Luminal BC

The high or low CD39 expression groups were determined by the optimal cut-off value (4.18) using ROC curve analysis. Chi-square test and Fisher’s exact tests were used to compare the differences in clinic pathological features between the two groups. Several clinicopathological characteristics demonstrated to be significantly associated with high CD39 expression (Table 2), such as patient age (*P* < 0.0001), N classification (N0 vs N2-N3-NX) (*P* = 0.0263).

**TABLE 2:** Correlations of CD39 expression in luminal BC tissues with clinicopathologic features.

Clinical characteristics	Variable	CD39 expression		Number of cases	χ^2^	*P*
		High *n* (%)	Low *n* (%)			
Ethnicity	HISPANIC OR LATINO	7 (3.98)	12 (7.02)	19 (5.48)	1.55	0.2133
	NOT HISPANIC OR LATINO	169 (96.02)	159 (92.98)	328 (94.52)		
Age	<60	130 (57.52)	100 (46.95)	230 (52.39)	4.92	**0.0266**
	≥60	96 (42.48)	113 (53.05)	209 (47.61)		
Histological type	Infiltrating ductal carcinoma	139 (61.50)	153 (71.83)	292 (66.51)	11.86	**0.0027**
	Infiltrating lobular carcinoma	67 (29.65)	34 (15.96)	101 (23.01)		
	Other	20 (8.85)	26 (12.21)	46 (10.48)		
ER	Negative	5 (2.21)	4 (1.88)	9 (2.05)	Fisher	1.0000
	Positive	221 (97.79)	209 (98.12)	430 (97.95)		
PR	Negative	26 (11.50)	38 (17.92)	64 (14.58)	3.61	0.0573
	Positive	200 (88.50)	174 (82.08)	374 (85.19)		
HER2	Negative	225 (99.56)	212 (99.53)	437 (99.54)	Fisher	1.0000
	Positive	1 (0.44)	1 (0.47)	2 (0.46)		
T classification	T1	63 (27.88)	54 (25.35)	117 (26.65)	Fisher	0.8713
	T2	125 (55.31)	121 (56.81)	246 (56.04)		
	T3	31 (13.72)	32 (15.02)	63 (14.35)		
	T4	7 (3.10)	5 (2.35)	12 (2.73)		
	TX	0 (0.00)	1 (0.47)	1 (0.23)		
M classification	M0	195 (86.67)	179 (84.43)	374 (85.58)	0.45	0.7982
	M1	2 (0.89)	2 (0.94)	4 (0.92)		
	MX	28 (12.44)	31 (14.62)	59 (13.50)		
N classification	N0	99 (43.81)	108 (50.70)	207 (47.15)	Fisher	**0.0327**
	N1	75 (33.19)	70 (32.86)	145 (33.03)		
	N2	29 (12.83)	24 (11.27)	53 (12.07)		
	N3	22 (9.73)	7 (3.29)	29 (6.61)		
	NX	1 (0.44)	4 (1.88)	5 (1.14)		
Stage	I	38 (17.04)	40 (18.87)	78 (17.93)	Fisher	0.8730
	II	124 (55.61)	123 (58.02)	247 (56.78)		
	III	57 (25.56)	46 (21.70)	103 (23.68		
	IV	2 (0.90)	2 (0.94)	4 (0.92)		
	X	2 (0.90)	1 (0.47	3 (0.69)		
Lymph node status	No	99 (43.81)	108 (50.70)	207 (47.15)	2.09	0.1478
	Yes	127 (56.19)	105 (49.30)	232 (52.85)		
Vital status	Alive	200 (88.50)	201 (94.37)	401 (91.34)	4.78	**0.0288**
	Dead	26 (11.50)	12 (5.63)	38 (8.66)		

*Values with “Not available,” “Not Evaluated,” “Unknown,” and “Indeterminate” were excluded.*

*Bold values of *P* < 0.05 indicate statistically significant correlations*

### High CD39 Is an Independent Prognostic Factor of Luminal BC

**FIGURE 4:**
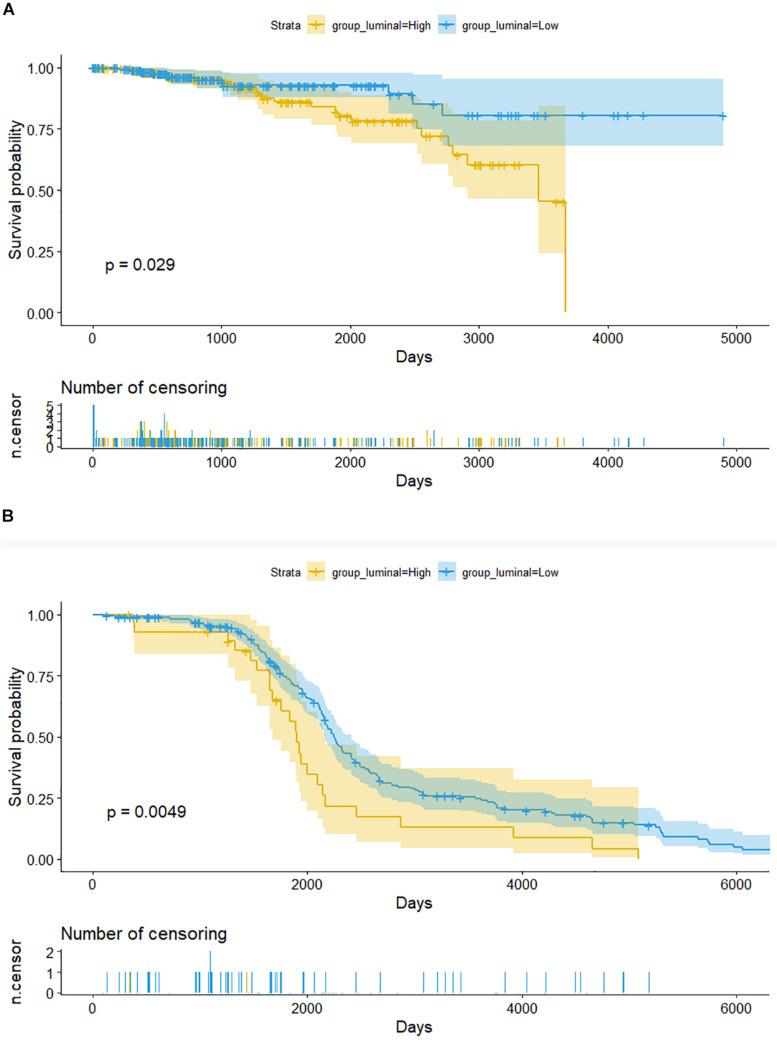
Kaplan–Meier curves of overall survival in luminal breast cancer patients. **(A)** TCGA cohort. **(B)** GSE86166 cohort.

We used Kaplan–Meier method to explore prognostic significance of CD39 expression in luminal BC patients. High CD39 was associated with poor OS in luminal BC patients (*P* = 0.029, Figure 4A). Validation using microarray datasets GSE86166 indicated similar prognostic prediction power of CD39 expression in BC (*P* = 0.0049, Figure 4B).

The relevant clinic pathological characteristics screened by multivariable analysis were used to assess the influence of CD39 expression on luminal BC patients, high CD39 was an independent prognostic factor associated with poor OS in the luminal molecular type subgroup (training: *P* = 0.0185; HR: 2.310, 95% CI: 1.151–4.637, validation: *P* = 0.0458; HR: 1.602, 95% CI: 1.009–2.543, Table 3).

**TABLE 3:** Multivariate analysis for luminal molecular type breast cancer patients using Cox regression.

Variable	HR (95% CI)	*P*-value
**Training (*N* = 439)**		
CD39 (high vs low)	2.310 (1.151, 4.637)	0.0185
N (N0 vs N2-N3-NX)	0.382 (0.164, 0.893)	0.0263
N (N1 vs N2-N3-NX)	0.449 (0.189, 1.071)	0.0710
Age (≥60 vs <60)	4.698 (2.295, 9.615)	<0.0001
Histological type (infiltrating ductal carcinoma vs other)	1.040 (0.391, 2.763)	0.9376
Histological type (infiltrating lobular carcinoma vs other)	0.630 (0.203, 1.956)	0.4239
**Validation (*N* = 247)**		
CD39 (high vs low)	1.602 (1.009, 2.543)	0.0458
Stage (2 vs 1)	0.939 (0.670, 1.316)	0.7142
Stage (3 vs 1)	0.954 (0.603, 1.510)	0.8402
Grade (2 vs 1)	1.044 (0.660, 1.651)	0.8532
Grade (3 vs 1)	0.757 (0.460, 1.247)	0.2744
Grade (4 vs 1)	0.226 (0.084, 0.609)	0.0033

### Gene Interactions and Enrichment Analysis of CD39

**FIGURE 5:**
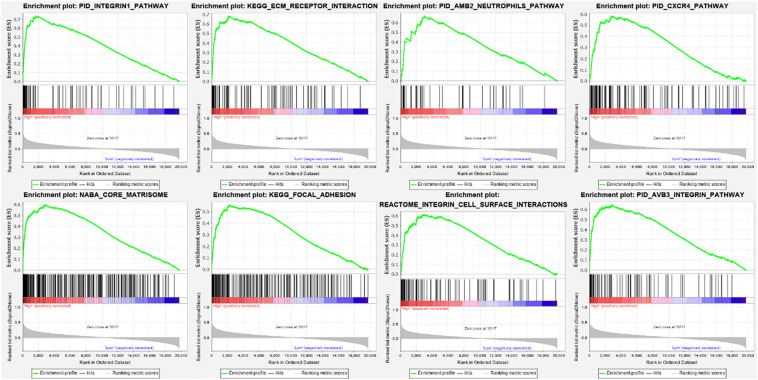
Enrichment plots from the gene set enrichment analysis (GSEA).

We conducted GSEA to find the different key signaling pathways between low and high CD39 expression datasets. Several significant different pathways (FDR < 0.05, normalized *P* < 0.05) were found in the enrichment of the MSigDB Collection (c2.cp.v6.2.symbols). Table 4 and Figure 5 demonstrated the most significantly enriched signaling pathways based on their normalized enrichment scores (NES). CD39 was related to the integrin1 pathway, ECM-receptor interaction pathway, AMB2-neutrophils pathway, CXCR4 pathway, core matrisome, focal adhesion, reactome integrin cell surface interactions, AVB3 integrin pathway.

**TABLE 4:** Gene sets enriched in CD39 phenotype high.

MSigDB collection	Gene set name	NES	NOM *P*-value	FDR *q*-value
C2.cp.v6.2.symbols.gmt	PID_INTEGRIN1_PATHWAY	2.223	<0.001	0.002
	KEGG_ECM_RECEPTOR_INTERACTION	2.229	<0.001	0.003
	PID_AMB2_NEUTROPHILS_PATHWAY	2.154	<0.001	0.004
	PID_CXCR4_PATHWAY	2.138	<0.001	0.004
	NABA_CORE_MATRISOME	2.158	<0.001	0.005
	KEGG_FOCAL_ADHESION	2.161	<0.001	0.005
	REACTOME_INTEGRIN_CELL_SURFACE_INTERACTIONS	2.163	<0.001	0.006
	PID_AVB3_INTEGRIN_PATHWAY	2.170	<0.001	0.007

### The Correlation Between Tumor-Infiltrating Immune Cells and CD39 Expression in Luminal Breast Cancer

**FIGURE 6:**
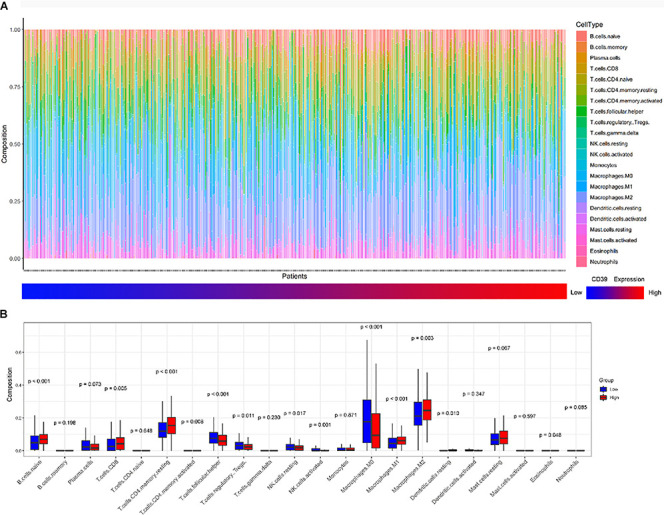
Correlations of CD39 expression with immune infiltration levels in the TCGA cohort. **(A)** The landscape of immune infiltration in 439 tumor tissues arranged by CD39 expression from low to high. **(B)** Analysis of differential immune cells between the low and high CD39 expression group in TCGA.

We then investigated whether CD39 expression influenced immune cell infiltration in BC. The CIBERSORT algorithm was performed and 439 luminal tumor samples in the TCGA cohort (total population = 1090) with a *P*-value < 0.05 were eligible for this study. Figure 6A demonstrated the immune infiltration landscape in BC obtained from 439 tumors arranged by CD39 from low to high. To further investigate the influence of CD39 on TIICs, CD39 within the last/top quarter was specified as the low/high group. There were significant intragroup and intergroup variations in the proportions of TIICs. The high CD39 group showed a higher fraction of CD8^+^ T (*P* = 0.005, Figure 6B) cells and macrophage M2 cells (*P* = 0.003, Figure 6B) than the low expression group. In contrast, the M0 macrophages fraction was relatively lower (*P* < 0.000, Figure 6B). Meanwhile, the high CD39 expression group consisted of a higher proportion of CD4^+^ memory resting T cells and naïve B cells, which had an immunosuppressive phenotype.

### The Correlation of Other Adenosine-Related Genes and Immune Checkpoints

CD39 plays an important role in producing adenosine. ENTPD1 encoding CD39 (*P* = 0.0009) was upregulated in BC tissues (Figure 3). Three ARs in BC tissues were then analyzed: ADORA2A, ADORA2B, and ADORA3 (GSE45827). The results indicated that ADORA2A (*P* = 0.0076) and ADORA3 (*P* = 0.0142) receptors were significantly upregulated in BC tissues (Figure 3), reported being associated with tumor immune escape. Correlation analysis revealed that there is a positive correlation between ENTPD1 and ADORA2A (*R* = 0.17, *P* = 0.041), ADORA3 (*R* = 0.23, *P* = 0.006).

## Discussion

We demonstrated that CD39 was higher in tumors compared to normal tissues from the TCGA data. CD39 expression was significantly associated with patient age, histological type, PR, and vital status. High CD39 was linked to poor luminal BC survival, and the multivariable Cox analyses confirmed that CD39 was an independent prognostic divisor in luminal BC using the TCGA cohort. GEO datasets validated the same results. CD39 expression has been identified to be associated with survival of luminal BC patients based on TCGA database mining for the first time.

It is known that in the tumor microenvironment, eATP can enhance immune responses and contribute to cancer cell death. CD39 can sequential hydrolysed eATP to AMP which can further degraded to anti-inflammatory adenosine by CD73 (Xiao et al., 2019). Recently, several CD39 antibodies had been developed in consideration of the potential value of CD39 as a therapeutic target (Bastid et al., 2013) mediated by reducing immunosuppressive adenosine or the increase in eATP (Moesta et al., 2020).

CD4^+^ T and CD8^+^ T cells has a pivotal role in cancer protective immunity. In BC, CD4^+^ T cells infiltration in the tumor microenvironment positively correlates with decreased OS (Matkowski et al., 2009). Our results revealed that the BC tissue with high CD39 expression had a higher scale of CD8^+^ T cells and M2 macrophages, however the M0 macrophages scale was relatively lower, suggesting that CD39 can promote TAMs toward M2 differentiation. Meanwhile, the high CD39 expression group comprised a higher proportion of naïve B cells and CD4^+^ memory resting T cells that presented immunosuppressive phenotypes, so CD39 is actually an exhaustion marker of T cells (Duhen et al., 2018; Simoni et al., 2018; Thelen et al., 2018). This is consistent with previous research, which revealed that more resting memory CD4^+^ T cells existed in ER+ cancers (Zhang et al., 2019).

The enrichment of CD4^+^ T cell infiltration have significant implications for prognosis (Zhang et al., 2019). CD39 may increase the resting memory of CD4^+^ cells by several mechanisms: (a) the function of anti-tumor T cells might be decreased by the adenosine generated by CD39; (b) CD39-generating adenosine can promote apoptosis of T-cells; (c) macrophages and dendritic cells can be polarized into immunosuppressive regulatory cells resulting in a T cells limitation; (d) A2A receptor activation induces T regulatory cells and myeloid-derived suppressor cells (MDSCs) cell expansion and increases the immunity suppression; (e) CD39-generating adenosine can interact with specific G-protein-coupled receptors-A1, A2A, A2B, and A3 which can weak anti-tumor immunity through enhance suppressive immune cells and attenuate the protective immune cells (Stagg and Smyth, 2010; Vigano et al., 2019). Deficient in the adenosine receptor A2A was related to the adenosinergic pathway in tumor immunity. Therefore, in this study, we performed an investigation into the genes’ expression on the adenosinergic pathway based on TCGA datasets. ADORA2A and ADORA3 expression were significantly upregulated in BC, positively correlated with high CD39 expression. Thus, an over activated CD39-adenosine axis might contribute to BC immune escape and progression.

The different molecular subtypes of BC, including HER2-enriched, basal-like, luminal A, and luminal B, showed different prognosis (Sorlie et al., 2003). Luminal B subtype occupied for nearly 40% of BCs (Metzger-Filho et al., 2013). Luminal B BC is characterized by a low ER expression, a low PR expression, and a high histological grade (Harbeck et al., 2013). It generally demonstrated an aggressive behavior and has a prognosis similar to the HER2-enriched and base-like subtypes (Tran and Bedard, 2011). CD39-adenosine axis overactivated can play a vital role in luminal BC through immune escape pathways.

Further studies are needed to validate the link between CD39 and tumor immunity. CD39 may be an effective therapeutic target in luminal BC, as long as it is confirmed by further fundamental and clinical studies.

This study also has several limitations. Firstly, the OS of BC patients may be impacted by the other risk factors which were not collected in TCGA database, such as tumor size, treatments et al. Secondly, luminal BC comprises of luminal A and luminal B subtypes which may have significantly different OS, but it was hard to separate according to the current TCGA database. Third, it is better to involve the same covariates in training and validation datasets, but unfortunately, age was not collected in the validation set.

## Conclusion

In the present analysis, CD39 expression correlates with the prognosis of luminal BC. This is the first study to demonstrate that CD39 is related to luminal BC survival based the immune pathway through TCGA database mining to the best of our knowledge. Further studies are warranted further to elucidate this potential novel therapeutic strategy for luminal BC.

## Data Availability Statement

The datasets presented in this study can be found in online repositories. The names of the repository/repositories and accession number(s) can be found in the article/supplementary material.

## Ethics Statement

The studies involving human participants were reviewed and approved by the Ethics Committee of Zhongshan Hospital, Fudan University. Written informed consent for participation was not required for this study in accordance with the national legislation and the institutional requirements.

## Author Contributions

XN and LH: conceptualization. WW: methodology. LH: software and data analysis. XN and XL: writing-original draft preparation. LH, ZH, JM, and WY: writing-review and editing. All authors have read and agreed to the published version of the manuscript.

## Conflict of Interest

The authors declare that the research was conducted in the absence of any commercial or financial relationships that could be construed as a potential conflict of interest. The reviewer YW declared a past co-authorship/collaboration with one of the authors LH to the handling Editor.

## Footnotes

1 https://cancergenome.nih.gov/

2 https://www.ncbi.nlm.nih.gov/geo/

3 https://cibersort.stanford.edu/index.php
